# The risk of venous thromboembolism and physical activity level, especially high level: a systematic review

**DOI:** 10.1007/s11239-020-02372-5

**Published:** 2021-01-02

**Authors:** H. Danin-Mankowitz, A. Ugarph-Morawski, F. Braunschweig, P. Wändell

**Affiliations:** 1 Division of Family Medicine and Primary Care, Department of Neurobiology, Care Sciences and Society (NVS), Huddinge, Karolinska Institutet, Alfred Nobels Allé 23 (D2), SE-141 83 Solna, Sweden; 2 Academic Primary Care Centre, Region Stockholm, Stockholm, Sweden; 3 Department of Medicine, Karolinska Institutet, Solna, Sweden; 4 Themes Heart and Vascular, Karolinska University Hospital, Solna, Sweden

**Keywords:** Physical activity, Venous thromboembolism, Deep venous thrombosis, Pulmonary embolism, Upper extremity venous thrombosis, Gender

## Abstract

Venous thromboembolism (VTE) is the third most common cause of cardiovascular disease. Connection between high level of physical activity (PA) and the onset of VTE is unknown. We searched the literature on the possible association between PA level, especially high levels, and the risk of VTE. A systematic review was carried out to identify relevant articles on the relation between PA level and VTE. The initial search was conducted together with the Karolinska Institutet University Library in February 2018, with follow-up searches after that. In total, 4383 records were found and then screened for exclusion of duplicates and articles outside the area of interest. In total, 16 articles with data on 3 or more levels of PA were included. Of these, 12 were cohort and 4 were case-control studies. Totally 13 studies aimed at investigating VTE cases primarily, while three studies had other primary outcomes. Of the 16 studies, five found a U-shaped association between PA level and VTE risk, although non-significant in three of them. Two articles described an association between a more intense physical activity and a higher risk of VTE, which was significant in one. Nine studies found associations between increasing PA levels and a decreasing VTE risk. Available literature provides diverging results as to the association between high levels of PA and the risk of venous thromboembolism, but with several studies showing an association. Further research is warranted to clarify the relationship between high level PA and VTE.

**Supplementary Information:**

The online version of this article (10.1007/s11239-020-02372-5) contains supplementary material, which is available to authorized users.

## Highlights

- Low physical inactivity (PA) is a risk factor for venous thromboembolism (VTE).
- Several studies but not all showed an association between high levels of PA and increased risk of VTE.
- More studies on association between PA and VTE risk with proper methods are needed.

## Introduction

Venous thromboembolism (VTE) is the third most common cause of cardiovascular disease, after coronary artery disease and stroke [1]. The annual incidence of VTE in Sweden is estimated at 150–200 per 100,000 person-years and the risk increases with age [2, 3]. In Sweden, over 11,000 patients are nursed annually in hospitals because of VTE, and around 40,000 medical visits within outpatient care are due to VTE [4].

VTE can express itself in various ways, from a completely asymptomatic thrombosis to a massive lung embolization with fatal outcome. Anticoagulant treatment is effective in preventing recurrence but can cause bleeding complications [5]. There are also models to predict recurrent VTE, above all the Vienna model [6], with a higher recurrent risk among men, patients with proximal DVT or PE, and higher D-dimer levels.

Physical activity and its positive effects on good health and well-being are well established. A total of 150 min of physical activity of at least moderate intensity per week, or at least 75 min of high intensity, is recommended by the Public Health Agency of Sweden which is consistent with that from the World Health Organisation (WHO) [7]. Low levels of physical activity are associated with an increased risk of cardiovascular morbidity [8]. As regards VTE, bed rest is a known risk factor for VTE, and a sedentary lifestyle also seems to be associated with an increased VTE risk [9–11].

Notwithstanding the positive health effects, there are also health risks associated with PA, in particular when strenuous exercise is concerned. The occurrence of cardiovascular events and also sudden cardiac death in, for example, long-distance running is documented [12]. In a Danish study on jogging, a U-shaped association between dose of jogging and all-cause mortality was described [13]. However, it is unclear whether this type of association also applies to PA and the risk of VTE. In particular, it is unclear whether highly intense exercise, as practiced today by a growing number of recreational athletes up to high age, is a protecting factor or a risk. Furthermore, it is unclear whether VTE contributes to the momentarily increased risk of cardiovascular events during highly intense exercise, and whether such risk is related to specific sports/modes of exercise. Two earlier reviews on the association between high and low levels of PA and VTE risk concluded, that higher PA level showed lower VTE risk [9, 11], but did explore the possible higher risk of VTE with highest PA levels.

The aim of this review was to assess current knowledge on the risk of venous thromboembolism in association with high levels of physical activity as described in the literature.

## Method

**Fig. 1:**
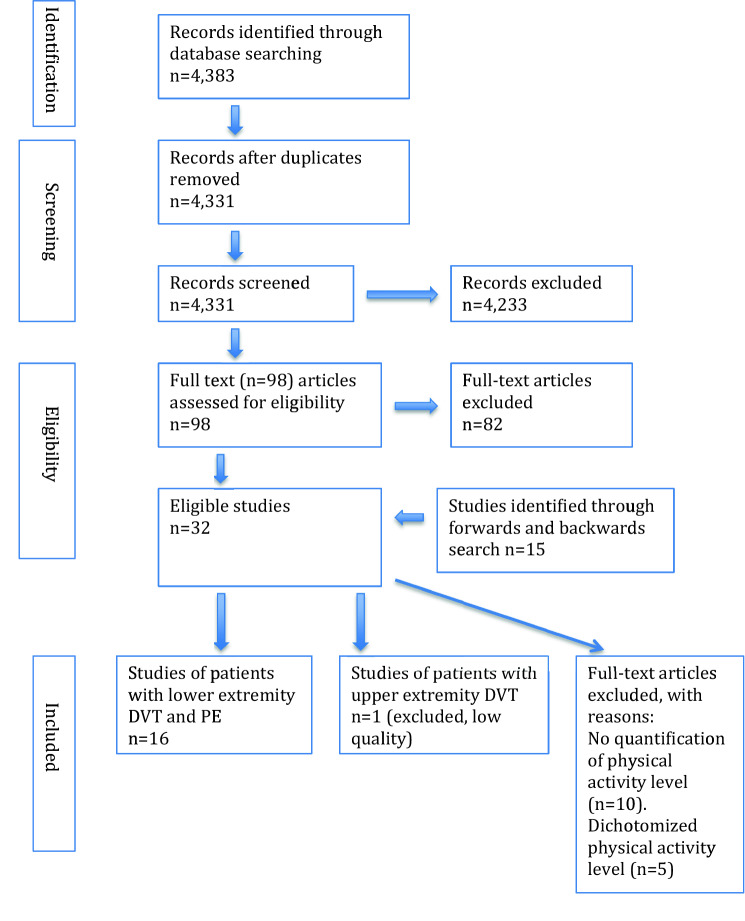
PRISMA 2009 flow diagram

Figure 1 describes the process of article inclusion. We searched without restrictions in terms of year, or publication type in the following databases: Medline (Ovid), Embase (embase.com), and Web of Science (Clarivate Analytics), to identify relevant articles and references. The searches were conducted by two librarians at the Karolinska Institutet University Library in February 2018. The complete search strategies are available as a supplementary file. The extensive search strategy included both free-text and MeSH terms and was initially created in Medline and later adapted to the other databases with corresponding vocabularies. Reference lists of included articles were also searched for finding relevant papers, and articles citing the already included studies were identified in further Google Scholar searches. In the end it remained 32 possible articles, which we have read carefully and finally included 16 articles in our systematic review. In additional search the last four articles were found [9, 14–16]. If in doubt whether an article would be included, HDM and PW discussed how to judge it.

An inclusion criterion was that physical activity (PA) should be categorized at least into three levels, in order to be able to quantify risk of VTE in strenuous activity level. Thus, 5 articles with only dichotomization of PA level were excluded, and besides another 11 not quantifying PA level at all were also excluded, leaving 16 articles left. A Flow chart on inclusion and exclusion of articles is shown in supplementary files.

We also assessed the quality of the review [17], as well as the included articles. In general, the included articles were of good quality according to both reviewers. As these were observational studies, both cohort and case-control studies were found and included. We excluded one article on venous thrombosis in the upper extremity owing to low quality, as it only showed the frequency of strenuous activity among men and associated incident thrombosis [18]. In the review by Evensen et al. original follow-up data from the Tromsø study was presented [9], i.e. 1994–2013, which extends results presented in an earlier article (1994–2007) [19]. We decided to include both these papers although review articles were otherwise excluded.

## Results

This systematic review included 16 articles (Table 1). Out of these, 12 were cohort and 4 were case-control studies. A total of 13 studies aimed at study VTE cases primarily, while in three other studies this was a secondary aim [20–22].

**Table 1:** Overview of included articles on the possible risk between high level of physical activity and venous thromboembolism

Main author	Country	Study design	Year performed	Study population (N)	VTE cases (n)	Follow-up	Ages	Men/women	Adjustments	Outcomes
Tsai 2002 [23]	US	Cohort (Atherosclerosis Risk in Communities study, ARIC; and Cardiovascular Health Study, CHS)	1987–1998	19,293	215 (1.45 per 1000 person-y)	Median follow-up 7.8y; 148,054 person-y	45–64 y (ARIC), ≥65 y (CHS) Mean age 59y at baseline	8660/10,633 (cases 113/102)	Sex, age, race, BMI, blood pressure, smoking, alcohol intake, education	Low physical activity (5 categories, high level > =2317(=ref), 1080- < 2317, 390- < 1080, 135- < 390,<135) CHS 1.00 no 95%CI. PA kcal/w.
Sidney 2003 [26]	US	Case-control	1998–2000	942 (196 cases/ 746 controls)	196		15–44 y Mean age cases: 35.3y, mean age controls 36.2y	Women	Age, race and ethnic groups, BMI, income, and VTE in the past	Vigorous physical activity (3 categories, no reg PA = ref) OR 0.50 (95% CI 0.35–0.72)
Glynn 2005 [1]	US	Cohort (Physician’s Health Study)	1982–2003	18,662	358 (Incident rate 10.9 per 10,000 person-y)	Median follow-up 20.1y; 329,526 person-y	40–84 y	Men	BMI, height, hypertension, elevated cholesterol, diabetes, current or former smoking, exercise, alcohol intake	HR 1.09 (95% CI 1.01–1.18) per one category increase (6 categories)
van Stralen 2007 [27]	The Netherlands	Population-based case-control (Multiple Environmental and Genetic Assessment of risk factors for venous Thrombosis, MEGA study)	1999–2004	7862 (3608 cases/ 4254 controls)	3608 (DVT 2093, PE 1044, DVT + PE 471)		18-70y	45.6%/54.4% of patients 46.7%/53.3% of controls	Sex, age, BMI, lifestyle factors and for matched/unmatched	Very strenuous sports activity (4 categories, no = ref) OR 0.66 (95% CI 0.56–0.76).
van Stralen 2008 [21]	US	Cohort (Cardiovascular Health Study, CHS)	1989–2001	5534	171	Median follow-up time 11.6y; 52,308 person-y	≥ 65y	2376/3158	Sex, age, race, self-reported health, BMI	U-shaped association. Strenuous exercise (4 categories, none = ref) HR 1.75 (95% CI 1.08–2.83)
Lindqvist 2009 [25]	Sweden	Cohort (Melanoma Inquiry of Southern Sweden, MISS)	1990–2002	29,518	312	Mean follow-up 11 y; 317,290 person-y	25–64 y	Women	Age, diagnosis of cancer during the study period, parity, smoking, alcohol intake, combined oral contraceptives (COC), exercise, BMI	Strenuous activity every week (3 categories, no = ref) HR 0.5 (95% CI 0.3–0.9)
Borch 2010 [19]	Norway	Population-based cohort (Tromsø study)	1994–2007	26,490	460 (DVT 295, PE 165) (1.61/1000 person-y)	Median follow-up 12.5 y; 286,467 person-y	25–97 y	12.598/ 13.892; cases 217/ 243	Age, sex, BMI, smoking, diabetes, and hormone therapy (women)	U-shaped, non-significant association. Physical activity (4 categories, 0 h = ref) ≥ 3 h/wk. HR 1.13 (95% CI 0.80–1.61)
Lutsey 2010 [29]	US	Cohort (Iowa Women’s Health Study)	1986–2004	40,377	2137 (DVT 1313, PE 824) (incidence rate 4.04 per 1000 person-years)	Median follow-up 13 y; 529,360 person-y	55–69 y; mean age 61.8 y.	Women	Age, BMI, educational attainment, smoking status, physical activity level	High physical activity (3 categories, low = ref) HR 0.91 (95% CI 0.82–1.02)
Wattanakit 2012 [22]	US	Community-based cohort (Atherosclerosis Risk in Communities study, ARIC)	1987–2005	15,340	468	Mean follow-up time 15.5y; 237,375 person-y	45-64y; mean age 54y	45% /55%	Sex, age, race, BMI, ARIC field centres	High physical activity (quartiles, lowest level = ref) HR 0.81 (95% CI 0.62–1.06); lowest risk at Q2 for both unprovoked and provoked VTE. Slightly U-shaped.
Bergendal 2012 [24]	Sweden	Case-control (Thrombo Embolism Hormone Study, TEHS)	2003–2009	2835 (1433 cases/ 1402 controls)	1433		18-64y	Women	Age, BMI, smoking, use of hormones, bed rest/minor trauma, surgery, cast, surgery and cast, prothrombin mutation and/or factor V, contraceptives	Strenuous activity (4 categories, light = ref) premenopausal OR 0.55 (95% CI 0.37–0.80). Postmenopausal OR 0.64 (95% CI 0.42–0.99).
Armstrong 2014 [20]	UK	Cohort (Million Women Study)	Scotland 1981–2008, England 1997–2012	1,119,239	14,550 (DVT 7712, PE ± DVT 7013)	Median follow-up 9y	50-64y; mean age 55.9y.	Women	Socioeconomic status, region, the first 4 y of follow-up, BMI, smoking, alcohol intake	U-shaped association. Strenuous activity (5 categories, rarely/never = ref) all VTE 1.08 (95% CI 0.99–1.17); DVT 1.13 (95% CI 1.01–1.27)
Olson 2015 [15]	US	Cohort (REasons for Geographic And Racial Differences in Stroke, REGARDS)	2003–2011	30,239	263 (DVT 153, PE ± DVT 122)	Median follow-up 5 y	≥45 years	45%/55%; case 153/110	Age, sex, income, education, race, region, and race*region interaction	Ideal physical activity (3 levels, poor = ref) HR 0.59 (95% CI 0.43–0.81)
Ogunmoroti 2016 [28]	US	Cohort (Multi-Ethnic study of Atherosclerosis, MESA)	2000–2015	6506	215 (3.3%) Event rates for poor, intermediate and ideal PA 4.0, 3.1 and 1.7 per 1000 person-y	Median follow-up 10.2 y	45–84 years	3074/3432; 215 cases	Age, sex, race/ethnicity, education, income	Ideal physical activity (3 levels, poor = ref) HR 0.70 (95% CI 0.52–0.95), intermediate 0.66 (95% CI 0.43–1.02)
Kim 2018 [16]	US	Case-control (NHS and NHSII, Nurses’ Health Study, HPFS, Health Professionals Follow-up Study)	1976–2014, 1989–2011, 1986–2012	6024 (2134 cases/3890 controls)	2134		30–55 y, 25–42 y, 40–75 y	Female, female, men F: 2450 (889 cases/1561 controls); +1766 (447 cases/1319 controls); M: 1808 (798 cases/1010 controls)	BMI, sitting time	ORs for poled data for MET quartiles in hr./wk.; Q1 (<5.6) ref., Q2 (5.6- < 15.4) 0.80 (0.69–0.94); Q3 (15.4- < 33.8) 0.84 (0.72–0.99); Q4 (33.8+) 0.71 (0.60–0.84)
Evensen 2018 [9]	Norway	Population-based cohort (Tromsø study)	1994–2013	26,490	754	19 y	25–97 y	12.598/ 13.892; cases	Age (as time scale), sex, BMI, and smoking	Slightly U-shaped, non-significant association. Hard physical activity (5 categories, 0 h = ref) > 3 h/wk. HR 1.11 (95% CI 0.83–1.49)
Johansson 2019 [14]	Sweden	Cohort (Venous thromboEmbolism In Northern Sweden, VEINS)	1985–2014	108,025	2054 (1.37 per 1000 person-y)	Median follow-up 15.5 y (1,496,669 person-years)	30–60 y; mean age 46.3 y	53,393/54,632; cases 1110/944	Age, BMI, hypertension, smoking, education level and cancer	Highest level ≥ 1–2 times/w (4 levels, never = ref) men 1.01 (95% CI 0.83–1.22), women 0.80 (95% CI 0.63–1.03)

Of the studies, two found a statistically significant U-shaped association between physical activity level and VTE risk [20, 21], while three studies showed a non-significant U-shape, i.e. two from the Norwegian Tromsø study [9, 19], and another by Wattankit et al. [22]. One study found an association between increased level of PA and a greater risk of VTE [1].

In the article by Tsai et al. [23], two measures of physical activity were applied because of two pooled cohorts, the ARIC (Atherosclerosis Risk In Communities) study used a score and the CHS (Cardiovascular Health Study) assessing kcal/week. We choose the latter measure allowing for a better discrimination of different PA levels. In the CHS cohort (≥ 65 years) an increased level of physical activity was non-significantly associated with an increased VTE risk. However, in the article by van Stralen et al. [21], using the same CHS cohort, a significant U-shaped association was found. Different cut-offs for physical activity level were used in the analyses, and the definition of strenuous activity seemed stricter in the article by von Stralen et al. [21], than in the study by Tsai et al. [23].

Seven studies found that an increasing physical activity level was associated with a decreasing VTE risk [15, 16, 24–28], and one showed a non-significant association [29]. One study found divergent results for men and women, with a non-significant lower risk among women but a similar risk as the reference group among men [2].

A special topic is the possible association between strenuous arm activity and VTE. Two studies showed a slightly elevated risk of upper extremity thrombosis with strenuous muscular activity of the upper extremity [18, 30], although both studies were non-significant. However, these studies were later excluded from the review because of the insufficient graded levels of PA.

## Discussion

The main finding of this systematic review is that the literature reports conflicting results regarding the association between different levels of PA and the associated risk of VTE, and especially regarding the potential risk of VTE with exercise on a high level.

While most of studies described a significantly decreasing VTE risk with increasing physical activity [15, 16, 24–28], also one with a non-significant association [29], another study found quite the opposite [1]. Some studies demonstrated or suggested a U-shaped association [9, 19–22]. One study found a different pattern between men and women [2], and one study found no association between PA level and VTE risk. However, as VTE may occur a long time after an exercise, a causal association and the identification of risk predictors may be difficult to establish.

Regarding levels of PA, this can be assessed in different ways, e.g. with the EPIC-PAQ suggested as suitable as a standardized measurement with 4 levels of PA [31]. For our concern a definition of strenuous levels of PA is warranted, and thus a more differentiated scale would be useful, e.g. the NOPAC with 10 categories [32], in order to be able to study the possible association between strenuous PA and incident VTE. In the study by Armstrong et al. [23], women reporting a strenuous exercise daily only comprised 3.2% of the population [33]. Categorizing samples into tertiles, quartiles or quintiles might not catch the group with the highest rate of strenuous physical activity.

There are possible mechanisms that could explain the association between strenuous PA and incident VTE. A review concluded, that long and vigorous extreme exertion causes hypercoagulability together with an augmented fibrinolysis [34], but the fibrinolytic parameters return to baseline quickly, whereas the procoagulant parameters remain elevated longer.

There are limitations with this study. Other reviews including a meta-analysis on the association between PA levels and VTE have been performed [11, 35]. However, these reviews compared low and high levels of PA in general regarding VTE risk, and not specifically very high PA levels in relation to VTE risk. We did not perform a meta-analysis, as the defined levels of PA differed largely across the studies, and especially the highest level of PA, with an obvious heterogeneity. The studies might not have identified a risk group with a very high level of physical activity and a higher VTE risk. The studies did measure physical activity level in different ways, why it could be hard to compare the different results. We decided to include different kind of studies, as our intention was to include all studies on the possible association between high levels of PA and incident VTE.

There are also several strengths. We performed a systematic search for relevant articles, and the findings in the earlier published review support that we have found relevant articles.

In conclusion, it is possible that high levels of PA could be a risk factor for VTE, but with the diverging results in the review the evidence for this is still unclear. More studies to analyse the association between high PA levels and VTE risk are needed, including an attempt to quantify possible risk level of high PA, and to try to use more standardized measurements of PA activity. Concerning the possible association between high level of PA and incident VTE a gap of evidence still remains.

## Supplementary Information

Below is the link to the electronic supplementary material.

Electronic supplementary material 1 (DOCX 65 kb)
