# Supplementary material for: The risk of venous thromboembolism and physical activity level, especially high level: a systematic review
Source: J Thromb Thrombolysis. 2021 Jan 2;52(2):508–16. doi: 10.1007/s11239-020-02372-5 (PMC8550020; doi:10.1007/s11239-020-02372-5)
Supplement: Supplementary file 1 — Electronic supplementary material 1 (DOCX 65 kb) [file 11239_2020_2372_MOESM1_ESM.docx]

Documentation of search strategies

University Library search consultation group

Date: Feb 2018

Topic/research question: Risk of venous thromboembolism associated with physical activity

Name of researcher(s): Per Wändell, NVS, Division of Family Medicine and Primary Care

Librarian(s): Sabina Gillsund & Magdalena Svanberg

Databases:

1. Medline (Ovid)
2. Embase (embase.com)
3. Web of Science (Clarivate Analytics)
4. ...

Total number of hits:

- Before deduplication: 5,942
- After deduplication: 4,383

Comments:

1. Medline

| Interface: Ovid  Date of Search: 13 Feb  Number of hits: 1,282  Comment: In Ovid, two or more words are automatically searched as phrases; i.e. no quotation marks are needed | Field labels   - exp/ = exploded MeSH term - / = non exploded MeSH term - .ti,ab,kf. = title, abstract and author keywords - adjx = within x words, regardless of order - * = truncation of word for alternate endings |
| --- | --- |
| 1. exp Venous Thrombosis/  2. exp Pulmonary Embolism/  3. exp Venous Thromboembolism/  4. (((venous or vein) adj3 thrombo*) or VTE or DVT or effort thrombo* or lung embol* or lung microembol* or lung thrombo* or paget schroetter syndrome or phlebothrombos* or pulmonary embol* or pulmonary microembol* or pulmonary thrombo*).ti,ab,kf.  5. or/1-4   6. Athletes/  7. exp Exercise/  8. Physical Exertion/  9. Physical Fitness/  10. exp Sports/  11. Dancing/  12. (exercise* or excertion or running or jogging or jumping or hopping or walk* or aerobic* or hiit or danc* or kung or power lifting or plyometric* or cardiopulmonary conditioning or isometric climbing or cycling or cyclist or horse riding or horse back riding or treadmill or aikido or alpinis* or archer* or athlete* or athletics or badminton or ballgame* or ball game* or baseball or basketball or biathl* or bicycle or bicycling or biking or bobsledder* or bobsleigh* or bowling or bowler* or boxing or boxer* or budo or canoe* or climbing or cricket or curling or decathl* or discus or diver or divers or diving* or dressage* or eventing or equestrian polo or fencer* or fencing or floorball or floor ball or football or golf* or gymnastic* or hammer throw* or handball* or handspring* or handstand* or headstand* or hepathl* or hockey or hurdler* or javelin throw* or judo or jujitsu or karate or kickbox* or kneeboard* or kung fu or lacrosse or luge or marathon* or martial art* or mountaineer* or paddleboard* or parathlet* or para-athlet* or para athlet* or pentathl* or pole vault* or powerlift* or race walk* or rafting* or racquetball or racketball or racket ball or regatta* or rider* or riding or rodeo* or rower* or rowing or rugby or running or runner* or sail* or shot put* or skateboard* or skater* or skating or skier* or skiing or snowboard* or soccer or softball or squash or sport* or sprinter* or sprinting or steeplechase* or swim* or tae kwon do or taekwondo or "track and field*" or triathl* or trampoline or tumbling or volleyball or water polo or weight* lifting or lifting weight* or wakeboard* or weightlifting or wrestl* or wushu).ti,ab,kf.  13. (physical* adj2 (fitness or condition or exertion or activ* or effort*)).ti,ab,kf.  14. ((strength* or resistance) adj2 (muscle* or program*)).ti,ab,kf.  15. ((aerobic or anaerobic or cardio* or condition or endurance or fitness or interval* or physical* or strength* or muscle or resistance) adj3 training*).ti,ab,kf.  16. ((high or long or triple or equestrian or show or ski) adj1 jump*).ti,ab,kf.  17. or/6-16  18. 5 and 17  19. Case Reports/ not (exp Review/ or exp Clinical Study/ or Multicenter Study/ or Meta-Analysis/)  20. 18 not 19  21. limit 20 to english  22. limit 21 to yr="1980 -Current"  23. remove duplicates from 22 | |

2. Embase

| Interface: embase.com  Date of Search: 13 Feb  Number of hits: 2,062  Comment: Emtree is the controlled vocabulary in Embase | Field labels   - /exp = exploded Emtree term - /de = non exploded Emtree term - ti,ab = title and abstract - NEAR/x = within x words, regardless of order - * = truncation of word for alternate endings |
| --- | --- |
| #1 'vein thrombosis'/exp #2 'venous thromboembolism'/exp #3 ((venous OR vein) NEAR/3 thrombo*):ti,ab #4 vte:ti,ab OR dvt:ti,ab OR 'effort thrombo*':ti,ab OR 'lung embol*':ti,ab OR 'lung microembol*':ti,ab OR 'lung thrombo*':ti,ab OR 'paget schroetter syndrome':ti,ab OR phlebothrombos*:ti,ab OR 'pulmonary embol*':ti,ab OR 'pulmonary microembol*':ti,ab OR 'pulmonary thrombo*':ti,ab #5 #1 OR #2 OR #3 OR #4  #6 'athlete'/exp #7 'exercise'/exp #8 'fitness'/exp #9 'sport'/exp #10 'dancing'/exp #11 exercise*:ti,ab OR excertion:ti,ab OR jogging:ti,ab OR jumping:ti,ab OR hopping:ti,ab OR walk*:ti,ab OR aerobic*:ti,ab OR hiit:ti,ab OR danc*:ti,ab OR kung:ti,ab OR 'power lifting':ti,ab OR plyometric*:ti,ab OR 'cardiopulmonary conditioning':ti,ab OR 'isometric climbing':ti,ab OR cycling:ti,ab OR bicycle*:ti,ab OR cyclist:ti,ab OR 'horse riding':ti,ab OR 'horse back riding':ti,ab OR treadmill:ti,ab OR aikido:ti,ab OR alpinis*:ti,ab OR archer*:ti,ab OR athlete*:ti,ab OR athletics:ti,ab OR badminton:ti,ab OR ballgame*:ti,ab OR 'ball game*':ti,ab OR baseball:ti,ab OR basketball:ti,ab OR biathl*:ti,ab OR bicycle:ti,ab OR bicycling:ti,ab OR biking:ti,ab OR bobsledder*:ti,ab OR bobsleigh*:ti,ab OR bowling:ti,ab OR bowler*:ti,ab OR boxing:ti,ab OR boxer*:ti,ab OR budo:ti,ab OR canoe*:ti,ab OR climbing:ti,ab OR cricket:ti,ab OR curling:ti,ab OR decathl*:ti,ab OR discus:ti,ab OR diver:ti,ab OR divers:ti,ab OR diving*:ti,ab OR dressage*:ti,ab OR eventing:ti,ab OR 'equestrian polo':ti,ab OR fencer*:ti,ab OR fencing:ti,ab OR floorball:ti,ab OR 'floor ball':ti,ab OR football:ti,ab OR golf*:ti,ab OR gymnastic*:ti,ab OR 'hammer throw*':ti,ab OR handball*:ti,ab OR handspring*:ti,ab OR handstand*:ti,ab OR headstand*:ti,ab OR hepathl*:ti,ab OR hockey:ti,ab OR hurdler*:ti,ab OR 'javelin throw*':ti,ab OR judo:ti,ab OR jujitsu:ti,ab OR karate:ti,ab OR kickbox*:ti,ab OR kneeboard*:ti,ab OR 'kung fu':ti,ab OR lacrosse:ti,ab OR luge:ti,ab OR marathon*:ti,ab OR 'martial art*':ti,ab OR mountaineer*:ti,ab OR paddleboard*:ti,ab OR parathlet*:ti,ab OR 'para athlet*':ti,ab OR pentathl*:ti,ab OR 'pole vault*':ti,ab OR powerlift*:ti,ab OR 'race walk*':ti,ab OR rafting*:ti,ab OR racquetball:ti,ab OR racketball:ti,ab OR 'racket ball':ti,ab OR regatta*:ti,ab OR rider*:ti,ab OR riding:ti,ab OR rodeo*:ti,ab OR rower*:ti,ab OR rowing:ti,ab OR rugby:ti,ab OR running:ti,ab OR runner*:ti,ab OR sail*:ti,ab OR 'shot put*':ti,ab OR skateboard*:ti,ab OR skater*:ti,ab OR skating:ti,ab OR skier*:ti,ab OR skiing:ti,ab OR snowboard*:ti,ab OR soccer:ti,ab OR softball:ti,ab OR squash:ti,ab OR sport*:ti,ab OR sprinter*:ti,ab OR sprinting:ti,ab OR steeplechase*:ti,ab OR swim*:ti,ab OR 'tae kwon do':ti,ab OR taekwondo:ti,ab OR 'track and field*':ti,ab OR triathl*:ti,ab OR trampoline:ti,ab OR tumbling:ti,ab OR volleyball:ti,ab OR 'water polo':ti,ab OR 'weight* lifting':ti,ab OR 'lifting weight*':ti,ab OR wakeboard*:ti,ab OR weightlifting:ti,ab OR wrestl*:ti,ab OR wushu:ti,ab #12 (physical* NEAR/2 (fitness OR condition OR exertion OR activ* OR effort*)):ti,ab #13 ((strength* OR resistance) NEAR/2 (muscle* OR program*)):ti,ab #14 ((aerobic OR anaerobic OR cardio* OR condition OR endurance OR fitness OR interval* OR physical* OR strength* OR muscle OR resistance) NEAR/3 training*):ti,ab #15 ((high OR long OR triple OR equestrian OR show OR ski) NEAR/1 jump*):ti,ab  #16 # 6 OR #7 OR #8 OR #9 OR #10 OR #11 OR #12 OR #13 OR #14 OR #15  #17 ('case report'/de OR 'case study'/de) NOT ('review'/exp OR 'clinical trial'/exp OR 'meta-analysis'/exp)  #18 #16 NOT #17  #19 [english]/lim  #20 [1980-2018]/py  #21 ('article'/it OR 'article in press'/it OR 'review'/it OR 'short survey'/it)  #22 #18 AND #19 AND #20 AND #21 | |

3. Web of Science Core Collection

| Interface: Clarivate Analytics  Date of Search: 13 Feb  Number of hits: 2,598 | Field labels   - TS/Topic = title, abstract, author keywords and Keywords Plus - NEAR/x = within x words, regardless of order - * = truncation of word for alternate endings |
| --- | --- |
| #1 TS=(((venous OR vein) NEAR/3 thrombo*) OR VTE OR DVT OR “effort thrombos*” OR “lung embol*” OR “lung microembol*” OR “lung thromboembol*” OR “paget schroetter syndrome” OR phlebothrombos* OR “pulmonary embol*” OR “pulmonary microembol*” OR “pulmonary thromboemboli*”)  #2 TS=(exercise* OR excertion OR jogging OR jumping OR hopping OR walk* OR aerobic* OR hiit OR danc* OR kung OR sport* OR "power lifting" OR plyometric* OR "cardiopulmonary conditioning" OR "isometric climbing" OR cycling OR bicycle* OR bicycling OR cyclist OR "horse riding" OR "horse back riding" OR treadmill OR aikido OR alpinis* OR archer* OR athlete* OR athletics OR badminton OR ballgame* OR "ball game*" OR baseball OR basketball OR biathl* OR biking OR bobsledder* OR bobsleigh* OR bowling OR bowler* OR boxing OR boxer* OR budo OR canoe* OR climbing OR cricket OR curling OR decathl* OR discus OR diver OR divers OR diving* OR dressage* OR eventing OR "equestrian polo" OR fencer* OR fencing OR floorball OR "floor ball" OR football OR golf* OR gymnastic* OR "hammer throw*" OR handball* OR handspring* OR handstand* OR headstand* OR hepathl* OR hockey OR hurdler* OR "javelin throw*" OR judo OR jujitsu OR karate OR kickbox* OR kneeboard* OR "kung fu" OR lacrosse OR luge OR marathon* OR "martial art*" OR mountaineer* OR paddleboard* OR parathlet* OR para-athlet* OR "para athlet*" OR pentathl* OR "pole vault*" OR powerlift* OR "race walk*" OR rafting* OR racquetball OR racketball OR "racket ball" OR regatta* OR rider* OR riding OR rodeo* OR rower* OR rowing OR rugby OR "running" OR "runner*" OR sail* OR "shot put*" OR skateboard* OR skater* OR skating OR skier* OR skiing OR snowboard* OR soccer OR softball OR squash OR sprinter* OR sprinting OR steeplechase* OR swim* OR "tae kwon do" OR taekwondo OR "track and field*" OR triathl* OR trampoline OR tumbling OR volleyball OR "water polo" OR "weight* lifting" OR "lifting weight*" OR wakeboard* OR weightlifting OR wrestl* OR wushu) OR TS=((physical* NEAR/2 (fitness OR condition OR exertion OR activ* OR effort*))) OR TS=(((strength* OR resistance) NEAR/2 (muscle* OR program*))) OR TS=(((aerobic OR anaerobic OR cardio* OR condition OR endurance OR fitness OR interval* OR physical* OR strength* OR muscle OR resistance) NEAR/3 training*)) OR TS=(((high OR long OR triple OR equestrian OR show OR ski) NEAR/1 jump*))  #3 #1 AND #2  #4 Refined by: **LANGUAGES:** ( ENGLISH ) AND **PUBLICATION YEARS:** ( 1989 OR 1999 OR 2009 OR 1990 OR 2000 OR 2010 OR 1991 OR 2001 OR 2011 OR 1992 OR 2002 OR 2012 OR 1993 OR 2003 OR 2013 OR 1994 OR 2004 OR 2014 OR 1995 OR 2005 OR 2015 OR 1982 OR 1996 OR 2006 OR 2016 OR 1985 OR 1997 OR 2007 OR 2017 OR 1986 OR 1998 OR 2008 OR 2018 OR 1988 ) AND **DOCUMENT TYPES:** ( ARTICLE OR BOOK CHAPTER OR REVIEW OR CORRECTION OR NEWS ITEM OR BIOGRAPHICAL ITEM OR RETRACTED PUBLICATION ) | |
